# Relationship between Angiotensin-Converting Enzyme Insertion/Deletion Polymorphism and the Risk of COVID-19: A Meta-Analysis

**DOI:** 10.1155/2023/3431612

**Published:** 2023-11-28

**Authors:** Hu Luoyi, Pan Yan, Fan Qihong

**Affiliations:** Department of Pediatrics, The First Affiliated Hospital of Yangtze University, Jingzhou, Hubei Province, China

## Abstract

**Introduction:**

Research shows the correlation between angiotensin-converting enzyme (ACE) deletion and insertion (D/I) polymorphism and COVID-19 risk; yet, conclusive evidence is still lacking. Thus, a meta-analysis of relevant articles was performed to more accurately estimate the relationship of ACE I/D polymorphism with the risk of COVID-19. *Material and Methods*. Relevant literature from the PubMed database was systematically reviewed, and odds ratios (ORs) and associated 95% confidence intervals (CIs) were measured. Additionally, the metapackage from Stata version 15.0 was used for statistical analysis.

**Results:**

The meta-analysis eventually contained 8 studies, including 1362 COVID-19 cases and 4312 controls. Based on the data, the ACE I/D polymorphism did not show an association with COVID-19 risk (D vs. I: OR = 1.25, 95% CI = 0.96–1.64; DD vs. II: OR = 1.89, 95% CI = 0.95–3.74; DI vs. II: OR = 1.75, 95% CI = 0.92–3.31; dominant model: OR = 1.88, 95% CI = 0.99–3.53; and recessive model: OR = 1.24, 95% CI = 0.81–1.90). Further, subgroup analyses stratified based on case proved that the ACE D allele demonstrated an association with increasing risk of COVID-19 severity (D vs. I: OR = 1.64, 95% CI = 1.01–2.66; DD vs. II: OR = 4.62, 95% CI = 2.57–8.30; DI vs. II: OR = 3.07, 95% CI = 1.75–5.38; dominant model: OR = 3.74, 95% CI = 2.15–6.50; and recessive model: OR = 1.28, 95% CI = 0.46–3.51).

**Conclusions:**

The ACE D allele was clearly related to an enhanced risk of COVID-19 severity. Hence, it is imperative to take into account the influence of genetic factors during the development of future vaccines.

## 1. Introduction

In December 2019, some cases in Hubei Province, China, presented with symptoms of fever, cough, and tachypnea [1]. Computed tomography (CT) examinations presented confluent and profuse pulmonary abnormalities, which were first suggested to be bacterial pneumonia-related symptoms [2]. Nevertheless, normal suspected etiological agents were not detected through viral nucleic acid and bacterial culture tests, such as Haemophilus influenzae, adenoviruses, and Streptococcus pneumoniae [2]. Thus, the reason for pneumonia remained unclear before the analysis of bronchoalveolar lavage fluid (BALF) samples, which revealed a novel pathogen that had a similar genetic sequence with betacoronavirus (*β*-CoV) B lineage [3]. In addition, this novel virus exhibited 96% genomic similarity to bat coronavirus RaTG13 and 80% to severe acute respiratory syndrome virus (SARS-CoV), but only 50% similarity to Middle East respiratory syndrome coronavirus (MERS-CoV) [1, 3]. Subsequent sequencing analysis categorized this virus in the Coronaviridae family. Later, in February 2020, the virus was known by the International Virus Classification Commission as severe acute respiratory syndrome coronavirus 2 (SARS-CoV-2) [4]. Additionally, the SARS-CoV-2-induced disease is referred to as coronavirus disease 2019 (COVID-19), and it later developed into the global pandemic. As of January 10, 2023, there have been more than 660,131,952 confirmed cases globally, including 6,690,473 deaths (according to the World Health Organization) [5].

The clinical manifestations of patients with COVID-19 vary greatly from asymptomatic infection to severe pneumonia that may cause respiratory failure and death [6]. The renin-angiotensin-aldosterone system (RAAS) was reported to play an important role in COVID-19 pathogenesis [7]. Angiotensin-converting enzyme-1 (ACE1) and angiotensin-converting enzyme-2 (ACE2) have a vital function in keeping the homeostasis of RAAS. The downregulation of ACE2 expression leads to increased angiotensin-II (Ang-II), which causes increased vascular permeability, pulmonary edema, and apoptosis of the bronchial alveolar epithelial cells [7]. Consequently, this contributes to lung injury and fibrosis [8].

The angiotensin-converting enzyme (ACE) deletion and insertion (D/I) polymorphism (rs4646994) is among the most common human (D) and (I) polymorphisms of the ACE gene in populations and possibly responsible for varying ACE levels. The DD genotype results in the highest plasma ACE level, the ID genotype causes an intermediate level, and the II genotype induces the lowest level. For example, the ACE D allele increases the level of ACE-1 but decreases that of ACE-2, leading to an increase in angiotensin-2 and pulmonary edema progression, by increasing microvascular permeability. This phenomenon intensifies the clinical course and prognosis of diseases like acute respiratory distress syndrome (ARDS) [9]. The protective impact of ACE-2 against acute pulmonary syndrome has been reported by experimental research, showing that angiotensin-2 stimulation provides a major mechanism for treating acute lung injuries. Similarly, the 30-day mortality among patients with ARDS who have the ACE DD genotype, compared with the ID or II genotypes, possibly reveals the clinical significance of these mechanisms [10].

Much research has studied the correlation between the ACE I/D polymorphism with COVID-19. Controversial results may arise from insufficient statistics caused by small sample sizes and ecogeographical differences [11]. Meta-analyses can overcome such constraints that are common in individual studies. Thus, a meta-analysis was performed to more accurately estimate the relationship of the ACE I/D polymorphism with the risk of COVID-19.

## 2. Materials and Methods

### 2.1. Literature Retrieval Strategy

The current work was performed in line with the Preferred Reporting Items for Systematic Review and Meta-Analysis (PRISMA) guidelines. In addition, the following search terms including “Angiotensin-converting enzyme or ACE”, “polymorphism or variant”, and “COVID-19” were utilized to search the PubMed database. An additional manual search of relevant studies was also conducted in the reference lists. With regard to duplicate studies, we enrolled the most updated version with the largest sample size.

### 2.2. Inclusion Criteria and Data Extraction

Studies that met the inclusion criteria were enrolled: (a) case-control studies evaluating the correlation of the ACE I/D polymorphism with the risk of COVID-19, (b) articles that contained odds ratios (ORs) as well as corresponding 95% confidence intervals (CIs) in accordance with genotyping data, and (c) articles that had clear case and control sources. Additionally, the study exclusion criteria are shown as follows: (a) non-case-control studies assessing the relationship of the ACE I/D polymorphism with the risk of COVID-19; (b) letters, editorial articles, meta-analyses, reviews, and case reports; (c) studies with no adequate or valuable raw data; and (d) duplicate studies [12].

### 2.3. Data Collection

Two investigators reviewed the included studies for obtaining data with the use of a uniform data form. Any disagreement between them was settled by mutual negotiation. In addition, the following data were retrieved: first author, publication year, region, case/control numbers and genotype frequencies, and the Hardy-Weinberg equilibrium (HWE) of controls.

### 2.4. Statistical Analysis

Stata version 15.0 (Stata Corporation, College Station, TX, USA) was employed for statistical analyses. The relationship between the ACE I/D polymorphism and the risk of COVID-19 was analyzed using ORs and corresponding 95% CIs. Additionally, *I*-squared (*I*^2^) statistics was applied for measuring heterogeneity. By using the Mantel-Haenszel approach, the fixed-effects model was employed in the case where there was no distinct heterogeneity in pooled ORs across different studies; otherwise, the DerSimonian and Laird approach was utilized, and the random-effects model was adopted. A sensitivity analysis was conducted by removing an individual study one at a time to explore the influence of each study on the pooled ORs. Furthermore, subgroup analyses stratified by race, severe cases, and HWE were also performed. The sensitivity analysis was completed by removing one single study each time to analyze the remaining data. Finally, publication bias was evaluated based on Begg's funnel plot. *P* < 0.05 was indicative of statistical significance in Begg's test.

### 2.5. Functional Predictions

Bioinformatic analysis was performed using HaploReg v4.1 (http://pubs.broadinstitute.org/mammals/haploreg/haploreg.php) to predict the role of the ACE I/D polymorphism [13].

### 2.6. Trial Sequential Analysis

Repeated significance testing and a higher random error risk might affect the meta-analysis. Trial sequential analysis (TSA) also promoted our conclusion robustness by predicting the statistical significance threshold and required information size. This study adopted 5% and 20% as type I and type II error significance levels, respectively, whereas 20% was set as the relative risk reduction. The sufficient evidence level was suggested in the case of a cumulative *Z*-curve entering the insignificant area or crossing the TSA boundary, which indicated no need for subsequent investigation. Data were processed with the use of TSA software (version 0.9.5.10 beta) [14].

## 3. Results

### 3.1. Eligible Studies

**Figure 1:**
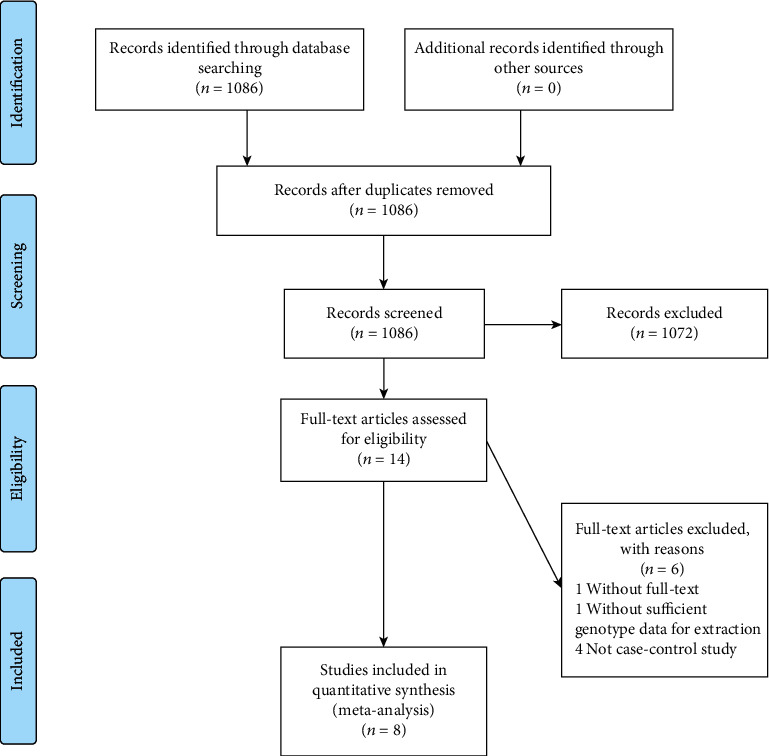
The flow diagram of included/excluded studies.

Figure 1 displays the study selection flowchart. Overall, there were 1086 related studies enrolled in the PubMed database. Finally, eight case-control studies published in English between 2020 and 2022 were recruited for the meta-analysis [15–22]. Tables 1 and 2 display the general characteristics of the eight studies enrolled. Notably, four articles were conducted in Caucasian populations, and the other four in Asian populations. In these articles, genetic distributions in the control groups were in line with HWE, except for Mohammad et al. and Elifcan et al.

### 3.2. Meta-Analysis Results

**Figure 2:**
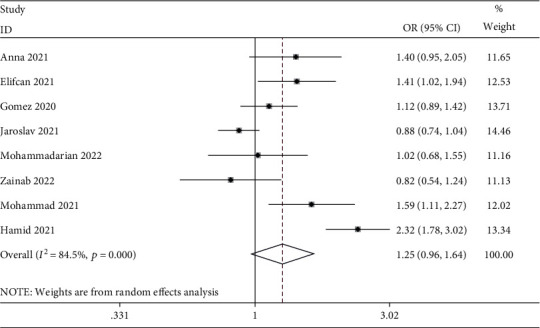
Forest plot for meta-analysis of the association between the ACE I/D polymorphism and COVID-19 risk using D vs. I. The solid diamonds and horizontal lines correspond to the study-specific ORs and 95% CIs. The gray areas reflect the study-specific weight. The hollow diamonds represent the pooled ORs and 95% CIs of the overall population. The vertical solid lines show the OR of 1, and the vertical dashed lines indicate the corresponding pooled OR.

Table 3 and Figure 2 present the correlation between ACE I/D polymorphism and the risk of COVID-19. According to our results, ACE I/D polymorphism did not demonstrate any clear relationship with COVID-19 risk with the use of diverse genetic models (D vs. I: OR = 1.25, 95% CI = 0.96–1.64; DD vs. II: OR = 1.89, 95% CI = 0.95–3.74; DI vs. II: OR = 1.75, 95% CI = 0.92–3.31; dominant model: OR = 1.88, 95% CI = 0.99–3.53; and recessive model: OR = 1.24, 95% CI = 0.81–1.90). As revealed by the race-stratified subgroup analysis, ACE I/D polymorphism did not show any relationship with COVID-19 risk in Caucasian or Asian populations. There was no significant difference in the stratified analysis on studies in accordance with HWE.

**Figure 3:**
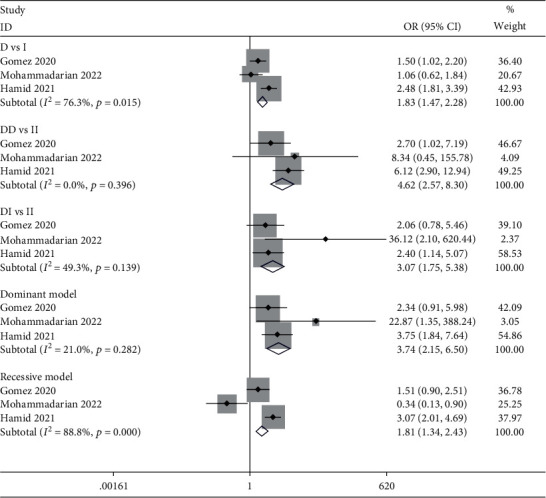
Forest plot for meta-analysis of the association between the ACE I/D polymorphism and COVID-19 severity risk using diverse genetic models.

The WHO guideline for the definition of disease severity was used to define nonsevere and severe cases [23]. Severe cases were those who had a positive result from a COVID-19 RT-PCR test, presented with clinical signs and severe pneumonia, and had any of the following conditions: severe respiratory distress, respiratory rate > 30 breath/min, or SpO_2_ < 90% in room air. Three articles were identified that met the severe case criteria [16, 18, 21]. Clearly, the ACE I/D polymorphism showed a significant relationship with the severity of COVID-19 (Figure 3 and Table 4, D vs. I: OR = 1.64, 95% CI = 1.01–2.66; DD vs. II: OR = 4.62, 95% CI = 2.57–8.30; DI vs. II: OR = 3.07, 95% CI = 1.75–5.38; dominant model: OR = 3.74, 95% CI = 2.15–6.50; and recessive model: OR = 1.28, 95% CI = 0.46–3.51).

### 3.3. Sensitivity Analysis and Publication Bias

**Figure 4:**
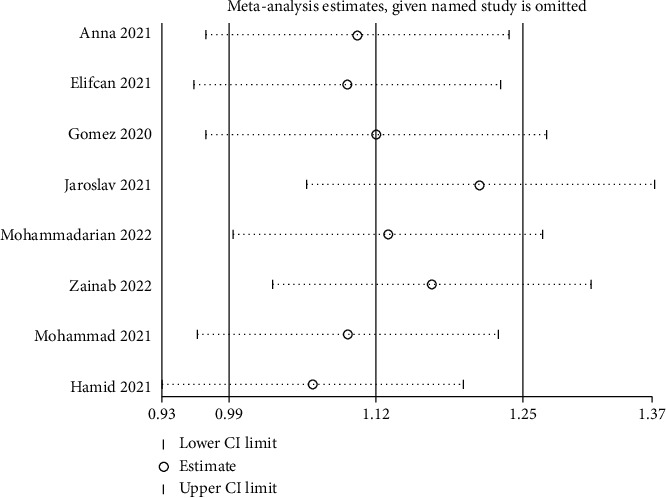
Sensitivity analysis of the association between the ACE I/D polymorphism and COVID-19 risk using D vs. I.

**Figure 5:**
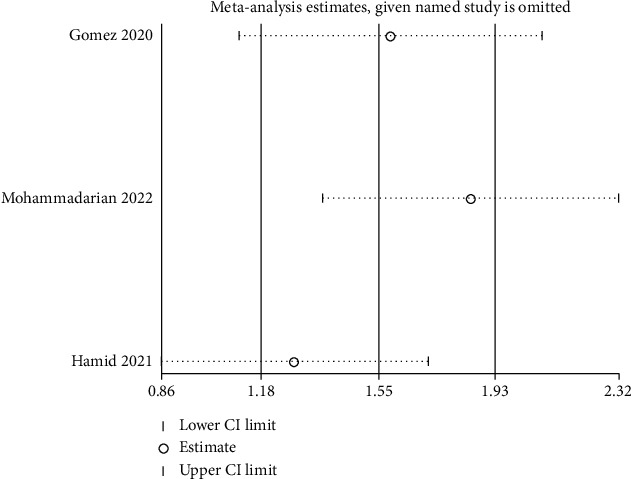
Sensitivity analysis of the association between the ACE I/D polymorphism and COVID-19 severity risk using D vs. I.

**Figure 6:**
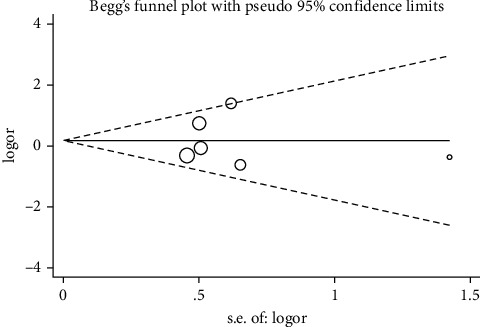
Begg's funnel plot analysis to detect potential publication bias for ACE I/D polymorphism with COVID-19 using D vs. I.

**Figure 7:**
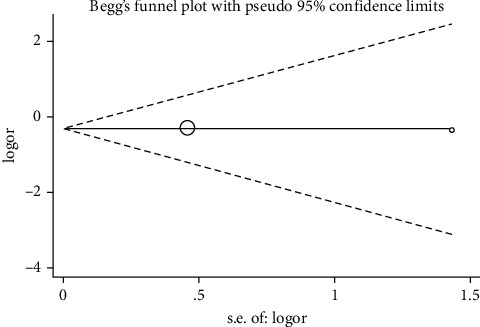
Begg's funnel plot analysis to detect potential publication bias for ACE I/D polymorphism with COVID-19 severity using D vs. I.

A sensitivity analysis was performed with the purpose of determining how each single study affected the pooled OR by eliminating an article each time. According to our results, none of the enrolled articles affected the pooled ORs, suggesting result stability (Figures 4 and 5). A Begg's test was conducted to evaluate publication bias, of which none was observed, implying no evidence of publication bias (Figures 6 and 7).

### 3.4. Functional Predictions

Data collected in HaploReg suggested no linkage disequilibrium of I/D polymorphism with additional variants of the ACE gene.

### 3.5. Trial Sequential Analysis

**Figure 8:**
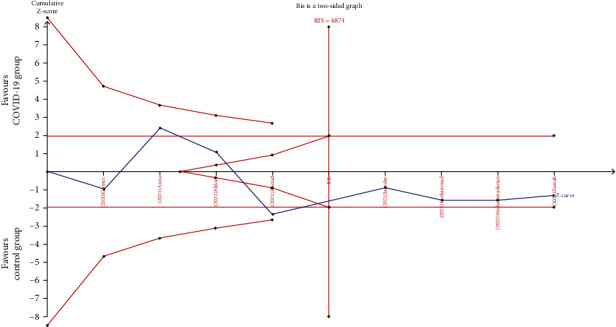
Trial sequential analysis of the ACE I/D polymorphism. The blue line represents the cumulative *Z*-score of the meta-analysis. The red straight line represents the conventional *P* = 0.05 statistical boundaries. RIS: required information size.

To reduce random errors and increase conclusion reliability, we performed TSA. As a result, no entering of cumulative *Z*-curves into futility or monitoring boundaries was observed, and the required information size was not reached (Figure 8). Therefore, our results revealed the nonrobustness of our conclusion, so the relationship of COVID-19 risk with the ACE I/D polymorphism should be further investigated.

## 4. Discussion

COVID-19 significantly threatens human health worldwide and induces an increased risk to public healthcare systems. Generally speaking, COVID-19 cases can be classified as asymptomatic, mild, or severe with ARDS, and such severe cases have an increased mortality rate because of stroke, respiratory failure, multiorgan failure, and thrombotic complications [23]. COVID-19 severity shows an increasing trend in patients with additional underlying diseases like diabetes, obesity, hypertension, or old age [24]. Nonetheless, numerous disease-free patients can experience ARDS or severe lung disease as well [25]. Therefore, the pathophysiological mechanism underlying COVID-19 remains largely unclear. Recently, ACE has been suggested to have a critical effect on acute lung disorders, especially ARDS [26]. Zainab et al. found that ACE I/D was not associated with the risk of developing COVID-19 [20]. However, Elifcan et al. found that the ACE I/D polymorphism could affect the clinical course of COVID-19 [16]. In addition, some studies found that the ACE I/D polymorphism could have the potential to predict the severity of COVID-19 [17, 18]. The inconsistency of results may be attributed to the different study designs, subject selection, or restricted statistical power [11]. Consequently, this meta-analysis is aimed at obtaining reasonable estimated results.

This meta-analysis was the first to summarize the existing data regarding the relation of ACE I/D polymorphism with COVID-19 susceptibility, which included 8 articles recruiting 1362 COVID-19 patients together with 4312 controls. Based on our findings, ACE I/D polymorphism did not show any significant relation with COVID-19 susceptibility. According to ethnicity-stratified subgroup analysis, no evident association was found among Caucasians or Asians. As revealed by subgroup analysis stratified by COVID-19 type, ACE D allele showed significant relation with the higher COVID-19 severity. Moreover, sensitivity analysis was also performed, revealing statistical robustness of our findings. In addition, the possible effect of ACE I/D polymorphism could be influenced by gene-gene interaction. But there are no studies of genetic polymorphisms that synergistically increase COVID-19 risk. Interaction between other risk factors and this polymorphism in relation to COVID-19 should be further studied.

This meta-analysis was the first to summarize existing data regarding the association of the ACE I/D polymorphism with COVID-19 risk, which included 8 articles with 1362 COVID-19 patients together and 4312 controls. Based on our findings, the ACE I/D polymorphism did not show any significant relationship with COVID-19 risk. According to ethnicity-stratified subgroup analysis, no evident association was found in Caucasian or Asian populations. As revealed by subgroup analysis stratified by COVID-19 type, ACE D allele showed a significant association with higher COVID-19 severity. Moreover, a sensitivity analysis was also performed, revealing the statistical robustness of our findings. In addition, the possible effect of the ACE I/D polymorphism could be influenced by gene-gene interaction. However, there are no studies of genetic polymorphisms that synergistically increase COVID-19 risk. Interaction between other risk factors and this polymorphism in relation to COVID-19 should be further studied.

The mechanism underlying the association between ACE I/D polymorphism and COVID-19 severity was not explored here. Nonetheless, research performed before the SARS-CoV-2 pandemic has suggested that the DD genotype is associated with morbidity and mortality in cases of ARDS [27]. Additionally, D allele frequency markedly increased among a hypoxemic group compared with a nonhypoxemic group, while the difference between control and SARS patients was not significant [28]. Based on prior database analyses, the frequency of the ACE II genotype within a population showed a remarkably negative association with mortality caused by SARS-CoV-2 infection, which indicated a favorable influence of the ACE II genotype on COVID-19 morbidity and outcome [29]. Moreover, Noel et al. reported in their systemic review that the ACE DD genotype was a potential marker that predicted the mortality risk in Asian COVID-19 cases with acute lung injury or ARDS [30]. Similarly, as indicated by Pati et al. in their epidemiological study of 26 Asian populations, the ACE D allele was related to SARS-CoV-2 mortality risk [31]. They also reported that the D allele of the ACE polymorphism was significantly related to COVID-19 severity and the D allele of the ACE1 I/D polymorphism was related to mortality risk.

There are some limitations in the current meta-analysis. First, this work only analyzed studies published in English, whereas those not published in English or those with unpublished data possibly satisfying our inclusion criteria were not included. Second, OR values were not corrected for age, race, or additional exposure factors associated with COVID-19 risk, which might affect outcome accuracy. Third, interactions between genes and between genes and the environment possibly affected outcome accuracy. Raw data were lacking, which made it impossible to evaluate these interactions further. Last, one individual gene set had limited diagnostic and predictive significance, which was thereby not recommended. According to data obtained from simulation studies as well as additional complex disorders, genetic profiling incorporating several genetic risk factors is promising in clinical use. Our genome-wide association research can shed more light on constructing the genetic risk profile of COVID-19. It is possible to predict COVID-19 by using the prediction model incorporating personal factors, genes, and environmental risk factors.

To conclude, the ACE D allele was clearly associated with an enhanced risk of COVID-19 severity. Previous research has demonstrated the significant influence of genetics on the immune response to vaccines. The extent to which genetic factors contribute to vaccine responses varies between 36.0% and 88.5% [32]. Gene polymorphisms have been found to have a discernible impact on vaccine immune response rates. By comprehending the functional and mechanistic effects of genetic polymorphisms, it may be possible to advance the development of novel vaccines. Gelder et al. conducted an association study examining the relationship between human leukocyte antigens (HLAs) and humoral immunity to influenza vaccinations. The impact of influenza vaccination on antibody levels varies among individuals based on HLA gene polymorphisms [33]. Further investigation is required to explore the potential beneficial role of the I/D polymorphism of ACE in the development of COVID-19 vaccines.

## Figures and Tables

**Table 1:** The included studies of ACE I/D polymorphism with COVID-19.

Study	Year	Country	Race	Cases/controls	Allele for cases		Allele for controls		Genotypes for cases			Genotypes for controls			HWE
					D	I	D	I	DD	ID	II	DD	ID	II	
Anna	2021	Greece	Caucasians	73/316	99	47	380	252	39	21	13	115	150	51	0.85
Elifcan	2021	Turkey	Caucasians	112/300	149	75	351	249	45	59	8	77	95	128	0.01
Gómez	2020	Spain	Caucasians	204/536	257	151	646	426	75	107	22	195	256	85	0.94
Jaroslav	2021	Czech	Caucasians	408/2579	301	317	2032	1878	91	210	107	701	1331	547	0.07
Mohammadarian	2022	Iran	Asians	91/91	104	78	103	79	17	70	4	33	37	21	0.10
Zainab	2022	Iraq	Asians	99/96	124	74	129	63	39	46	14	41	47	8	0.28
Mohammad	2021	Saudi Arabia	Asians	117/150	158	76	170	130	57	44	15	60	50	40	0.01
Hamid	2021	Iran	Asians	258/244	377	139	263	225	144	89	25	70	123	51	0.82

HWE: Hardy-Weinberg's equilibrium.

**Table 2:** The included studies of ACE I/D polymorphism with COVID-19 severity.

Study	Year	Country	Race	Cases/controls	Allele for cases		Allele for controls		Genotypes for cases			Genotypes for controls			HWE
					D	I	D	I	DD	ID	II	DD	ID	II	
Gómez	2020	Spain	Caucasians	67/536	93	41	646	426	31	31	5	195	256	85	0.94
Mohammadarian	2022	Iran	Asians	37/91	43	31	103	79	6	31	0	33	37	21	0.10
Hamid	2021	Iran	Asians	152/244	226	78	263	225	84	58	10	70	123	51	0.82

**Table 3:** Summary of different comparative results of ACE I/D polymorphism with COVID-19.

Variables	*N*	OR (95% CI)				
		D vs. I	DD vs. II	DI vs. II	Dominant model	Recessive model
Total	8	1.25 (0.96-1.64) R	1.89 (0.95-3.74) R	1.75 (0.92-3.31) R	1.88 (0.99-3.53) R	1.24 (0.81-1.90) R
Race						
Asians	4	1.35 (0.83-2.18) R	2.05 (0.89-4.72) R	1.96 (0.80-4.81) R	2.08 (0.96-4.49) R	1.16 (0.50-2.69) R
Caucasians	4	1.14 (0.90-1.45) R	1.79 (0.66-4.85) R	1.59 (0.59-4.26) R	1.72 (0.66-4.48) R	1.27 (0.80-2.03) R
HWE						
Yes	6	1.18 (0.85-1.65) R	1.40 (0.70-2.79) R	1.23 (0.70-2.17) R	1.36 (0.77-2.43) R	1.12 (0.65-1.93) R
No	2	1.49 (1.17-1.89) F	4.63 (1.20-17.83) F	4.63 (1.04-20.52) R	4.65 (1.11-19.49) R	1.68 (1.20-2.34) F

*N*: number; CI: confidence interval; OR: odds ratio.

**Table 4:** Summary of different comparative results of ACE I/D polymorphism with COVID-19 severity.

Variables	*N*	D vs. I	DD vs. II	OR (95% CI)	Dominant model	Recessive model
				DI vs. II		
	3	1.64 (1.01-2.66) R	4.62 (2.57-8.30) F	3.07 (1.75-5.38) F	3.74 (2.15-6.50) F	1.28 (0.46-3.51) R

## Data Availability

Data related to this paper can be made available from the corresponding author upon reasonable request.
